# Identifying the effectiveness of 3D culture systems to recapitulate breast tumor tissue *in situ*

**DOI:** 10.1007/s13402-023-00877-8

**Published:** 2023-09-30

**Authors:** Katarzyna A. Ludwik, Frances R. Greathouse, Samuel Han, Kimberly Stauffer, David R. Brenin, Thomas P. Stricker, Deborah A. Lannigan

**Affiliations:** 1 Department Pathology, Microbiology & Immunology, Vanderbilt University Medical Center, Nashville, TN 37232 USA; 2 PeerNova, San Jose, CA 95110 USA; 3 Department Surgery, University of Virginia, Charlottesville, VA 22908 USA; 4 Department Biomedical Engineering, Vanderbilt University, Nashville, TN 37235 USA

**Keywords:** Breast cancer, Heterogeneity, Chemotherapy, Organoid, HER1, Estrogen receptor alpha, Microenvironment

## Abstract

**Purpose:**

Breast cancer heterogeneity contributes to chemotherapy resistance and decreased patient survival. To improve patient outcomes it is essential to develop a technology that is able to rapidly select the most efficacious therapy that targets the diverse phenotypes present within the tumor. Breast cancer organoid technologies are proposed as an attractive approach for evaluating drug responses prior to patient therapy. However, there remain challenges in evaluating the effectiveness of organoid cultures to recapitulate the heterogeneity present in the patient tumor in situ.

**Method:**

Organoids were generated from seven normal breast and nineteen breast cancer tissues diagnosed as estrogen receptor positive or triple negative. The Jensen-Shannon divergence index, a measure of the similarity between distributions, was used to compare and evaluate heterogeneity in starting tissue and their resultant organoids. Heterogeneity was analyzed using cytokeratin 8 and cytokeratin 14, which provided an easily scored readout.

**Results:**

In the in vitro culture system HER1 and FGFR were able to drive intra-tumor heterogeneity to generate divergent phenotypes that have different sensitivities to chemotherapies.

**Conclusion:**

Our methodology, which focuses on quantifiable cellular phenotypes, provides a tractable system that complements omics approaches to provide an unprecedented view of heterogeneity and will enhance the identification of novel therapies and facilitate personalized medicine.

**Supplementary Information:**

The online version contains supplementary material available at 10.1007/s13402-023-00877-8.

## Introduction

Breast cancer death rates have decreased during the last several decades, but breast cancer is still the second leading cause of cancer deaths in women. In the clinic, breast cancer is screened for the presence of estrogen receptor (ER), progesterone receptor (PR) and amplification of ERBB2/HER2 (HER2). Based on these assessments tumors are divided into ER + , HER2 + with or without ER + , and triple negative breast cancer (TNBC) subgroups, and this categorization has been used to identify treatment options [1]. Molecular studies are also now used to inform treatment and provide targeted therapies [2]. However, a significant clinical problem is that inter- and intra-tumor heterogeneity limits therapy response [3–8]. Therefore, to improve breast cancer outcomes, in vivo and in vitro models need to recapitulate this heterogeneity in order to identify more effective treatments.

Patient-derived xenografts (PDX) have demonstrated an ability to predict patient response to treatment [9]. However, the establishment of PDX models requires substantial time and tumor evolution in the patient may differ from that in the mouse [10]. Two-dimensional cultures of dissociated human tumors do not recapitulate the structural complexity, cellular phenotypes, or gene expression profiles of the intact tumor tissue [11]. Three-dimensional culture systems with properties similar to the tissue or tumor of origin are an attractive alternative, and they have been particularly well characterized for the normal human gastrointestinal tract [12] and the normal human breast [13]. However, there is a substantial challenge in using organoids derived from tumor tissue to ensure that the heterogeneity has been successfully recapitulated. Organoid systems for colon cancer are currently the best validated for their relevance to the starting tumor tissue [14]. In contrast, the ability of breast cancer organoids to recapitulate the starting tissue has been very limited [15]. In some cases no comparison between the starting tumor tissue and the tumor organoid was attempted [16, 17]. Discordance between organoids and their starting tissue have been observed and these differences are amplified during in vitro passaging raising concerns over the physiological relevance of the organoids [18, 19]. Therefore, ideally, to ensure that meaningful clinical information can be obtained from organoid analysis, a simple method to aid in evaluating how effectively the organoids recapitulate the starting tissue would be useful for the breast cancer field.

This study identifies a method that readily evaluates the fidelity of the organoid system to recapitulate the inter- and intra- tumor heterogeneity of a particular patient’s breast tumor. For these studies a simple culture system was used to assess the effectiveness of the organoids to recapitulate the inherent heterogeneity in primary TNBC or ER + breast cancer. This culture system included amphiregulin (AREG) and fibroblast growth factor 7 (FGF7), which were identified as essential components for generating normal breast organoids from human tissue [13]. Additionally, AREG and FGF7 are necessary for mammary stem cell maintenance and are associated with breast cancer [20–23]. Cellular phenotype was used as a readout for analyzing tumor heterogeneity and the response of the tumor to known chemotherapeutic agents. Support for analyzing cellular phenotype as a readout is provided by the observations that the various cell types comprising the tumor have been shown to respond differentially to therapies [24]. To facilitate the comparison between the tumor tissue in situ and the organoid cultures we used the Jensen-Shannon divergence (JSD) method. The JSD method measures the similarity between the starting tissue and the organoids by calculating the distance between their probability distributions. To provide context for the JSD method we analyzed > 5, 684 images taken from starting tissue and organoid cultures obtained from nineteen different breast cancer patients and seven normal breast tissues.

To simplify the phenotypic approach, tumor heterogeneity was analyzed using cytokeratin 8 (K8) and cytokeratin 14 (K14) although the JSD method can be used with different biomarkers. Keratins are cytoplasmic intermediate filament proteins that are expressed in epithelial cells. The biomarkers K8 and K14 were selected based on their previous use as diagnostic markers for luminal breast cancer and TNBC, respectively [25]. K8 is expressed in the luminal cells of the normal breast and in breast cancer is correlated with a less invasive phenotype and increased overall survival [26, 27]. Loss of K8 is associated with a worse prognosis [28]. K14 is expressed in myoepithelial cells in the normal breast but is accepted as reliable marker of basal-like breast cancer (BLBC) [29, 30]. Approximately 70% of TNBCs are classified as BLBCs and this tumor type has the worst prognosis [31]. K14 has been correlated with a motile phenotype [32] [33–35] and the proliferation marker, Ki67 [36]. In contrast, reduced expression of K14 was correlated with longer relapse-free survival [37]. The JSD method provides a quantitative assessment of the heterogeneity within the starting tissue and the resultant organoids that are not readily defined by standard statistical methods due to the fact that the organoid population is an aggregate of distinct phenotypes.

This study clearly illustrates the challenge in representing the heterogeneity in an organoid model and also the necessity for developing approaches to quantitatively determine whether an organoid culture has recapitulated the tumor in situ. The JSD method succeeds in providing a quantitative approach and it can be used as a guide to further improve organoid cultures to better recapitulate the starting tumor tissue. Furthermore, enrichment of therapy-resistant populations in response to clinically-relevant drug treatments was easily identified using the JSD method. In summary, the JSD analysis provides a simple approach, which can be combined with other methodologies, to achieve the goal of personalized approaches to drug responses in breast cancer.

## Results

### Breast tumor heterogeneity

Tumor subtypes were based on clinical assessment and a pathologist independently evaluated the tissue used in this study (Fig. S1 and Table S1). The levels of ERα and EGF receptor (HER1), as appropriate for the tumor type, were also evaluated as a complement to the H&E sections. The data set contained tumors from nineteen different breast cancer patients and seven samples from breast surgery reduction (normal). To identify a method that will simplify the visualization and quantitation of intra- and inter-tumor heterogeneity we used immunofluorescence (IF) and initially, examined the distribution of K8 and K14 within the starting tissue (ST). K8 and K14 are expressed in the normal breast epithelium but alterations in the expression pattern of these markers occurs during malignant transformation in the breast and impacts patient survival [38]. Therefore, we initially analyzed how the expression of K8 and K14 correlated with normal breast tissue, ER + breast cancer or TNBC.

**Fig. 1:**
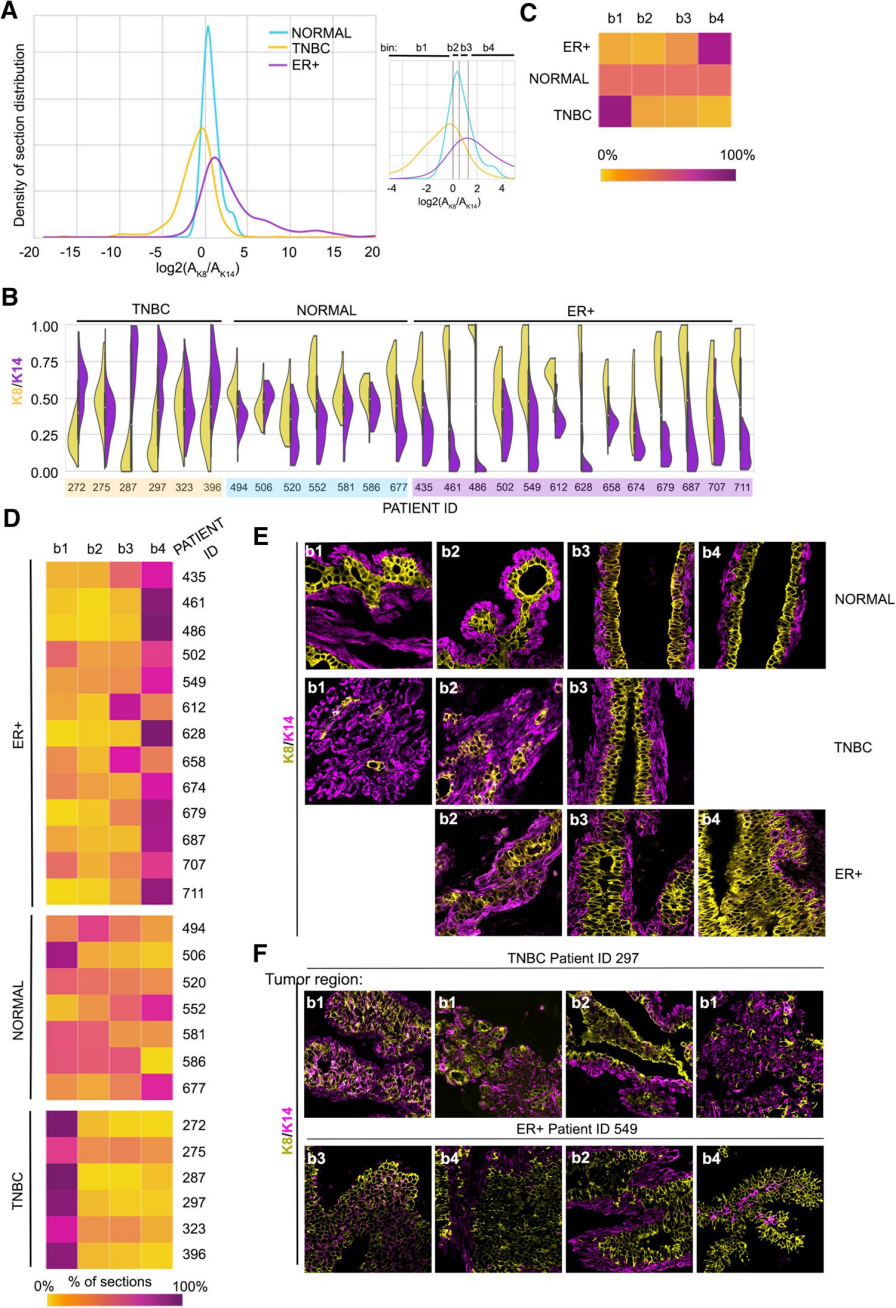
Probability density distribution as a method to visualize intra- and inter-tumor heterogeneity. **A** The log_2_ ratio of K8 + to K14 + areas plotted as a probability density distribution for normal breast tissue, TNBC, and ER + . The lines in the inset and the associated bin number indicate the quartiles associated with the normal tissue distribution. **B** Violin plots of the ratio of K8 + to K14 + areas for individual patient samples. **C** Heat map showing inter-tumor heterogeneity of all the starting tissue analyzed, which was based on the percentage of sections that fall within a defined bin. **D** Heat map showing intra- and inter-tumor heterogeneity of all the starting tissue analyzed, which was based on the percentage of sections that fall within a defined bin. **E** Representative images from the bins associated with a particular phenotype are shown for normal breast tissue, TNBC and ER + . **F** Images of sequential regions from individual tumors are shown to illustrate intra-tumor heterogeneity. See Tables 1, S1 and S2 and Fig. S1

Samples were taken from various random locations within the normal breast epithelium or breast cancer epithelium tissue to minimize sample bias. Formalin-fixed paraffin-embedded (FFPE) samples were prepared, and the sections analyzed for K8 and K14 by IF. The extent of K8 and K14 per area for each section was determined. The ratio of K8 + area to K14 + area (K_8_/K_14_) allowed us to compare two variables at the same time. Multiple sections were obtained for a patient’s starting tissue (ST), resulting in a total data set of 2,532 images obtained from the 26 starting tissues. The log_2_(K_8_/K_14_) was performed to facilitate data visualization. The distribution of TNBC skewed to the left of the normal reflecting the high K14 + content whereas the ER + breast cancer skewed to the right indicative of the high K8 + content (Fig. 1A). To further visualize heterogeneity, we plotted the K_8_/K_14_ ratio using violin plots to clearly illustrate the phenotype frequency distribution and highlight the heterogeneity of the individual samples (Fig. 1B). Normal breast tissue shows heterogeneity but histologically normal breast tissue in the ST or organoids is readily distinguished from tumor tissue by its evident apical-basal polarity. Taken together, these data illustrate the difficulties in evaluating whether the ST has been successfully recapitulated in the organoid culture. We argue that it is important to capture the diverse cellular phenotypes within the tumor because different cell types respond differently to therapies and may be a source of resistance [24, 39–41].

### Comparison of clinical phenotypes using Jensen Shannon divergence

To simplify the analysis of heterogeneity we generated four bins based on the quartile distribution of the log_2_(K_8_/K_14_) obtained in analysis of the normal tissue (Fig. 1A and inset). For each ST subtype the images were assigned to the bins based on the log_2_(K_8_/K_14_) and the section distributions were plotted as heatmaps. The number of organoids analyzed was limited by the amount of ST and/or the success of culturing. To ensure that we were accurately represent the underlying heterogeneity of the samples we calculated the minimal number of sections necessary to analyze. This calculation was based on the bin with the lowest frequency of sections for both the ER + and TNBC ST in our data set. The average frequency of the least represented bin for both distributions was ~ 0.08 (Table S2). Therefore, assuming that we sample the underlying population N-times, the probability of not obtaining a section in that cluster is (0.92)^N^. Thus, to obtain at least one section from that bin with 0.85 confidence a minimum of 23 sections needs to be analyzed, based on the formula 1-(0.92)^N^ = 0.85. This analysis was relevant to both ST and organoids (Tables S2 and S3). In most cases we exceeded the minimum sample size necessary to evaluate heterogeneity.

The aggregate heat map for the ER + ST shows an enrichment in K8 + cells whereas TNBC ST has more K14 + cells and by definition the normal distribution is present equally in all four bins (Figs. 1A, 1C). A heat map with the distribution for each starting tissue clearly illustrates the intra- tumor heterogeneity for patients with the same diagnosis (Fig. 1D). Representative images from each bin are shown for normal, TNBC and ER + tumor tissue (Fig. 1E). For example, patient samples 628, 679 and 711 have fewer sections in bin 1 than the other ER + breast tumor samples and patient sample 272 has fewer sections in bin 4 than the other TNBC samples. Representative images of sections through a single patient tumor clearly supports the variation captured in the heat maps (Fig. 1F). Collectively this straightforward analysis of K8 and K14 staining illustrates the intra- and inter-tumor heterogeneity that needs to be recapitulated within the organoid population if meaningful information on drug responses, which could translate to the patient, are to be obtained.

To generate a quantitative description of the intrinsic tumor heterogeneity we used the Jensen-Shannon divergence (JSD) method to compare the probability density distributions, as defined by the bins, within the ST and the organoids (ORG) generated from the ST based on the following formula:

$$JSI(P|\left|Q\right)=0.5(\sum P\left(logP-logM\right)+\sum Q(logQ-logM))$$

in which M = 0.5(P + Q), P = probability distribution of the ST sections from an individual patient X (X = patient identifier), Q = probability distribution of the ORG sections generated from the patient. Inherently JSD is asymptotically bound by zero and one, with one defining no similarity between distributions P and Q and 0 defining identical distributions. The general application of JSD assumes no specific limitation on the interrogated data. However, in case of our data, there are three major limitations to consider: (i) we are comparing to real data sets, ST and ORG, as opposed to real and simulated; (ii) the data does not assume infinite distribution possibilities as all of the data are generated from the breast and therefore, some distributions are not possible; and (iii) distributions sum to one as the data is represented by the percentage of the total. Due to these limitations, the boundaries of the JSD indexes obtained from our comparisons are narrowed. Therefore, to better represent and interpret JSD values resulting from our comparisons, we determined the most disparate JSD value that we could obtain from our data set, which was achieved by comparing ER + and TNBC ST (Tables S4-S9). By performing this comparison a JSD of 0.56 was obtained indicating that there are inherent similarities between these distinct tumor subtypes most likely because all the samples originate from the breast. To account for these underlying similarities the data were normalized such that 0.56 represents zero similarity. In these comparisons the higher the normalized JSD score the more similar samples are to each other. The normalized JSD score indicates that samples from patients diagnosed with the same clinical diagnosis are more similar to each other than patients with a different diagnosis (Table 1).

**Table 1:** Global JSD scores for comparisons between starting tissues of different subtypes. ST_1: subtype 1; ST_2: subtype 2 for a given comparison. Bold: the most disparate conditions ER + vs TNBC comparison used for JSD normalization. See Tables S4-S9

ST_1	ST_2	JSD	JSD_Norm
Normal	Normal	0.33	40.62
ER +	ER +	0.32	43.48
TNBC	TNBC	0.25	55.36
Normal	TNBC	0.39	30.71
Normal	ER +	0.40	29.09
ER +	TNBC	**0.56**	**-0.13**

**Fig. 2:**
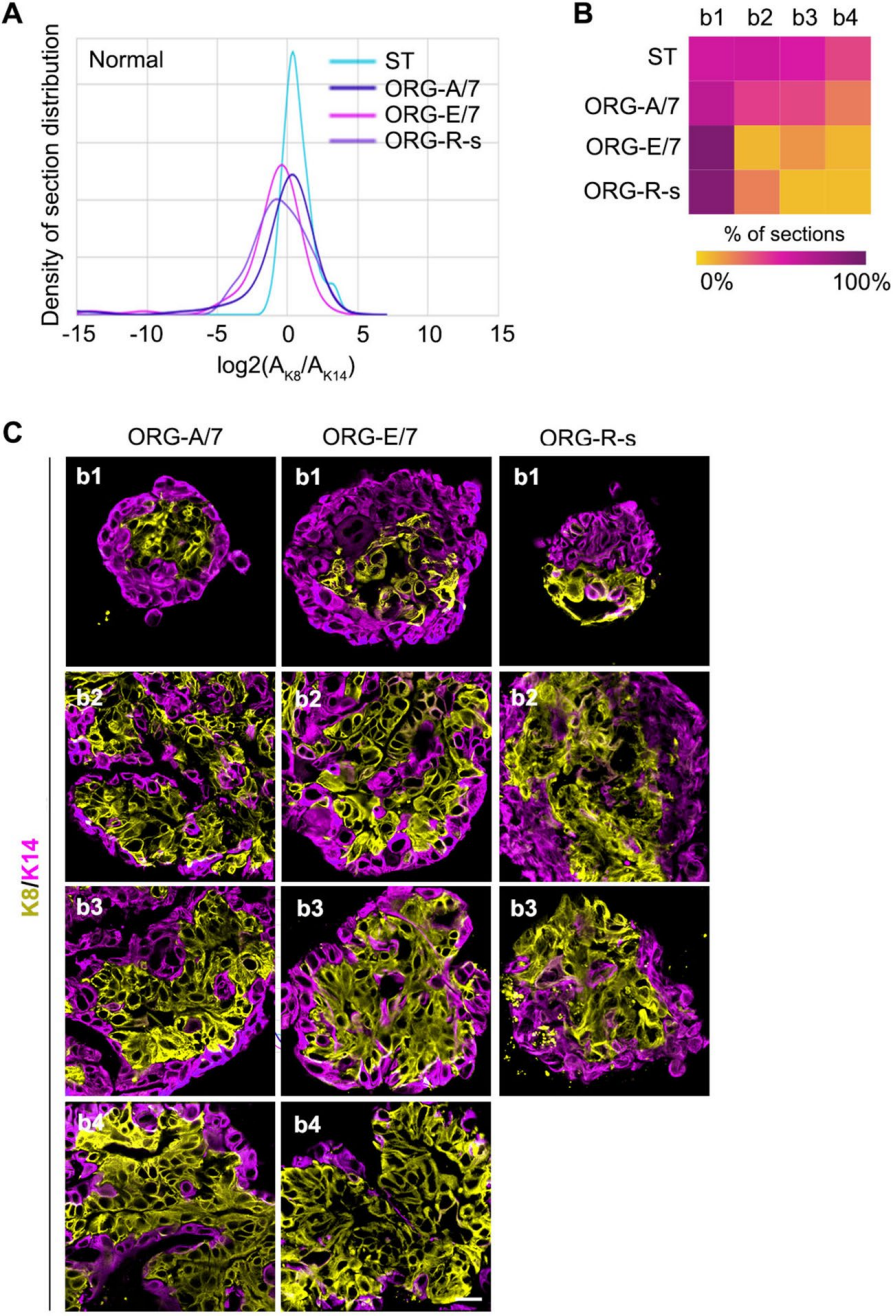
Probability density distribution is able to identify culture-induced phenotypic changes within the tissue. **A** The probability density distribution for starting tissue obtained from patients with a normal diagnosis and the resulting organoids cultured under various conditions. Base media was supplemented with AREG and FGF-7 (A/7), EGF and FGF-7 (E/7) or the R-spondin (R-S) media in which EGF was replaced with AREG (R-s). **B** Heat map showing the bin distribution for each culture condition. **C** Representative images from the bins associated with the culture conditions are shown. For A7 and E7 (N = 3 patients with > 87 organoid sections); (for R-S (N = 2 patients with 62 organoid sections). See Table 2

To demonstrate that the JSD is sensitive to treatment-induced changes in K8 + and K14 + distribution organoids were generated from normal breast tissue [13] and cultured in AREG/FGF7, EGF/FGF7, or a system based on intestinal organoid culture, referred to as the R-spondin (R-s) culture method [42]. AREG/FGF7 was chosen because previously we identified that this cocktail effectively recapitulated the normal ductal architecture in situ compared to EGF/FGF7, which caused abnormal expansion of the K14 + population [13]. The R-s culture method comprises a complex mixture that includes EGF. Therefore, based on our previous observations using normal breast tissue we replaced EGF with AREG. The distribution and representative images for each bin generated for the various culture conditions are shown (Figs. 2A-C). The JSD values were calculated similar to the equation above except that Q was defined as the probability distribution of organoid sections generated from a particular culture condition. For four out of five individuals the normalized JSD value for normal organoids cultured with AREG/FGF7 was ~ 80 compared to EGF/FGF7 and R-s, with a value of ~ 53 and ~ 29, respectively (Tables 2 and S3). The heat map illustrates that both the R-s and EGF culture conditions resulted in an enrichment of K14 + cells. The R-s culture method did not contain EGF, and therefore, other components of the R-s cocktail are responsible for the increase in K14 + cells. Taken together, these data highlight the importance of having a quantitative approach to analyzing whether the organoids have recapitulated the tissue in situ.

**Table 2:** Global JSD scores for comparison between normal starting tissue and organoids generated from normal tissue under varying conditions. Condition_1: starting tissue (ST); Condition_2: organoid culture condition (A7 amphiregulin and FGF7; E7 EGF and FGF7; R-s R-spondin with amphiregulin instead of EGF). See Table S3

Subtype	Condition_1	Condition_2	JSD	JSD_Norm
Normal	ST	A7	0.11	79.69
Normal	ST	E7	0.26	53.06
Normal	ST	R-s	0.40	29.17
TNBC	ST	A7	0.09	83.92
TNBC	ST	E7	0.13	76.80

### Analysis of intra- and inter-tumor heterogeneity using Jensen Shannon divergence

**Fig. 3:**
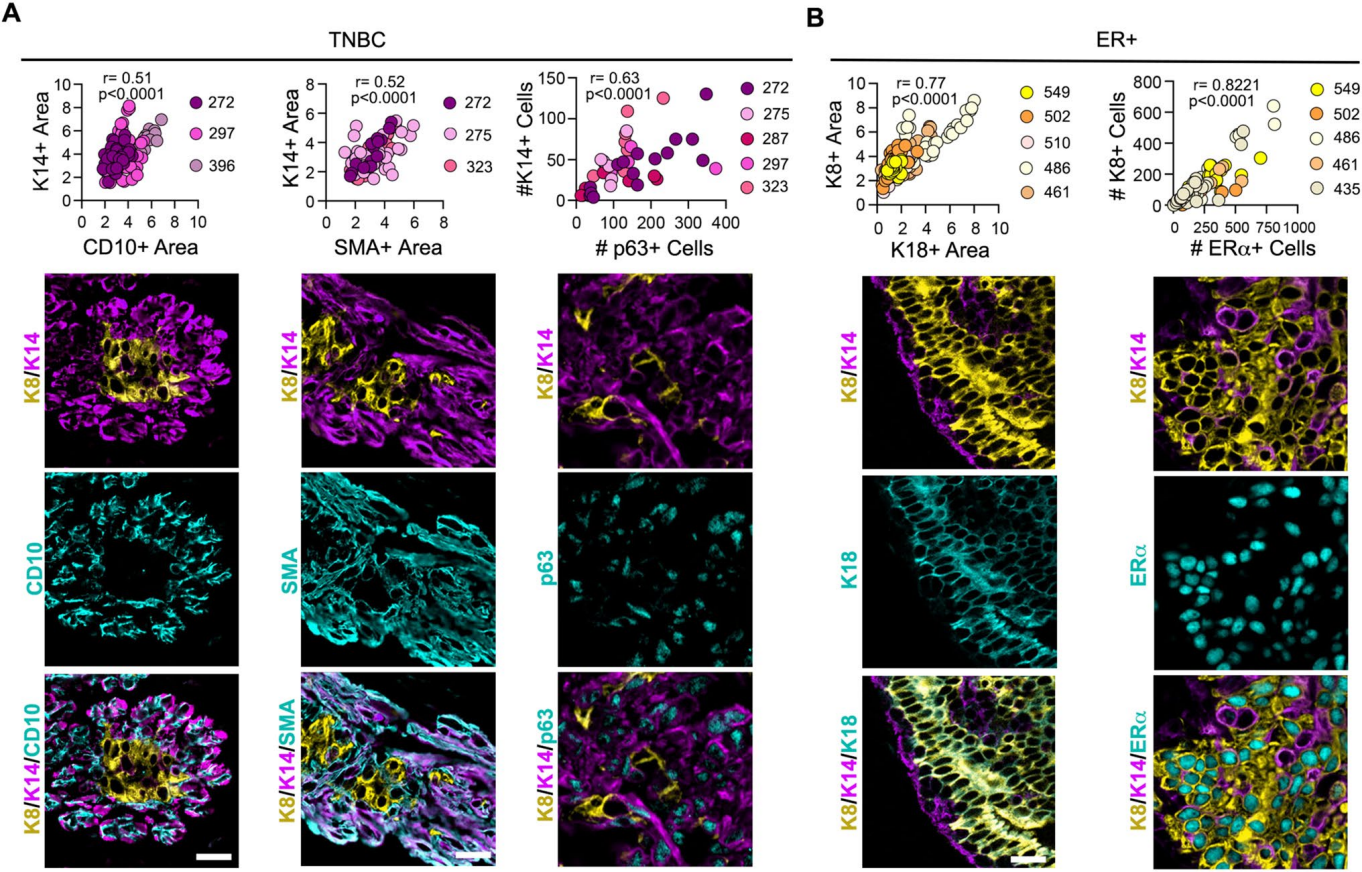
Identification of a minimal marker set to identify heterogeneity in breast cancer. **A** A positive linear correlation was observed between the areas of CD10 and SMA areas versus K14 + and the number of K14 + versus p63 + cells in TNBC. Representative images for each analysis are shown. The patient identification is indicated in the figure and each data point represents a section image with > 150 sections for each stain pair. **B** A positive linear correlation was observed for areas of K18 + versus K8 + and the number of K8 + versus ERα + cells in ER + breast cancer. Representative images for each analysis are shown. The number of patients is indicated in the figure and each data point represents a section image with > 70 sections for each stain pair

We also evaluated whether markers in addition to K8 and K14 would aid in characterizing tumor heterogeneity. For TNBC ST we analyzed cluster of differentiation 10 (CD10), smooth muscle actin (SMA) and p63 in combination with K14. As an aggregate of all samples analyzed the distribution of CD10, SMA and p63 compared to that of K14 shows a strong positive linear correlation, which was significant and indicates that these markers provided similar information to K14 (Fig. 3A). We next investigated the use of the markers K18 and ERα in ER + breast cancer in combination with K8 for the aggregate of all samples analyzed. The distribution of K18 and ERα generated a very strong positive correlation, which was significant with K8, demonstrating that K18 and ERα are redundant with K8 (Fig. 3B). This analysis shows that the distribution of K8 + and K14 + cells are able to assess tumor heterogeneity without increasing the complexity of the analysis by addition of other markers.

### Microenvironment contributions to the tumor phenotype

**Fig. 4:**
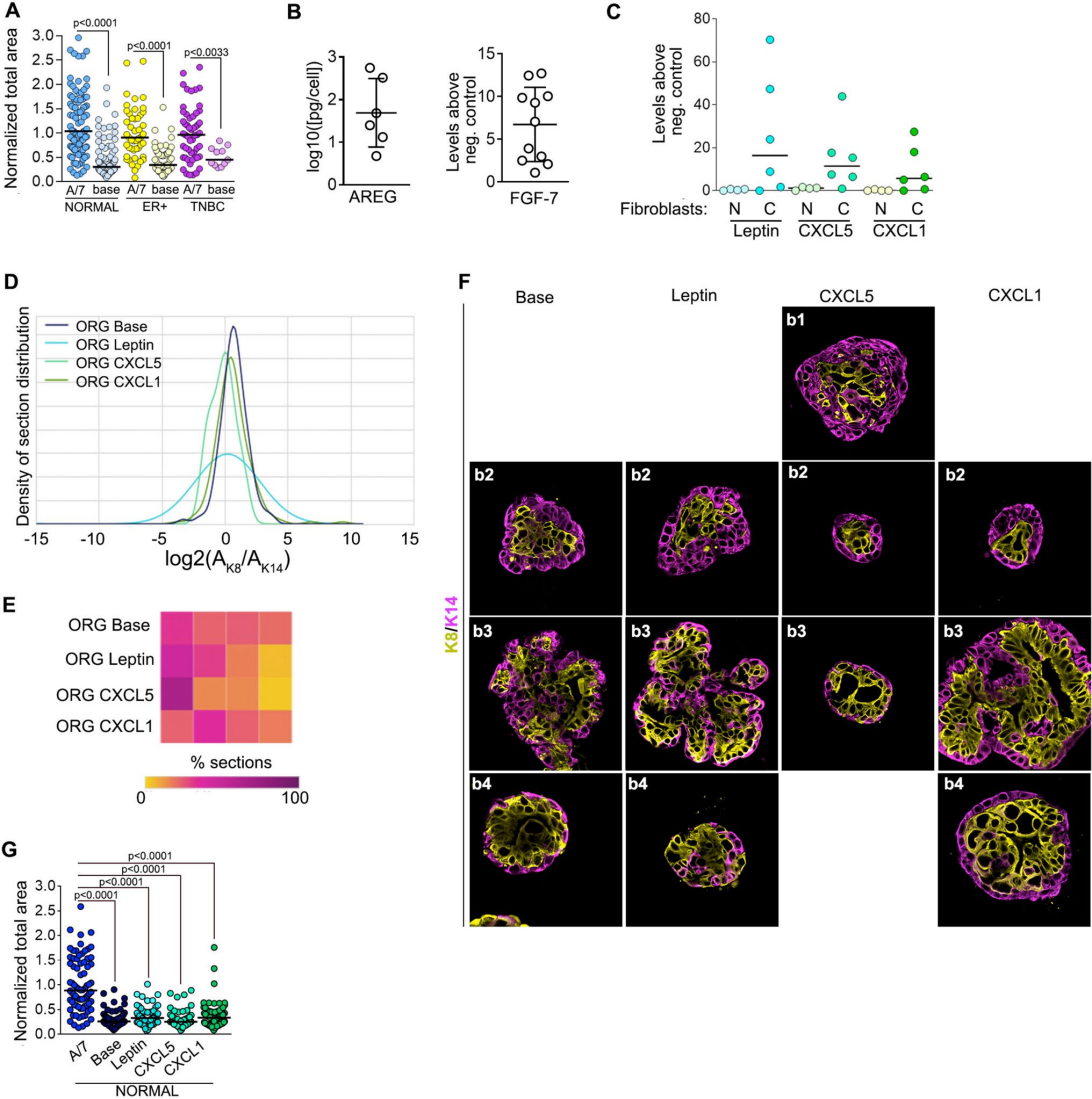
Fibroblast contribution to the breast microenvironment. **A** Normal and tumor organoid growth is dependent on the microenvironment. The area was determined by DIC and normalized to the area obtained in the A/7 culture media. **B** Normal and cancer-associated fibroblasts (CAFs) secrete FGF-7 and AREG. Each point represents a different patient sample. **C** CAFs secrete higher levels of leptin, CXCL1 and CXCL5 than normal fibroblasts. Each point represents a different patient sample. **D** CXCL5 increases the K14 + population in organoids generated from normal breast epithelium as determined by the probability density distribution. N ≥ 2 patients with ≥ 22 organoid sections analyzed for each condition. **E** Heat map showing the bin distribution for each culture condition. **F** Representative images of organoids generated from normal breast tissue cultured in the indicated cytokine are shown. **G** Leptin, CXCL1 and CXCL5 do not increase proliferation in organoids generated from normal breast epithelium. See Figures S2, S3 and Tables S3 and S10

In base media both normal and tumor organoids fail to expand, indicating that the epithelial tissue relies substantially on the microenvironment for its growth as opposed to autocrine signaling (Fig. 4A). To empirically identify optimal culture conditions, we performed growth factor and cytokine analysis from isolated normal and cancer-associated (CAF) stromal fibroblasts. All fibroblasts secreted FGF-7 and AREG, which we previously found necessary to generate proper ductal structure in organoids derived from the normal breast (Fig. 4B). The levels of EGF were ~ 500 fold below its K_d_ for HER1 and therefore, were not considered physiologically relevant. Furthermore, addition of EGF to TNBC, which has a high percentage of K14 + cells, resulted in an even greater increase in the K14 + population, which also argues against the use of EGF in organoid cultures (Fig. S2). The stromal fibroblasts secreted numerous cytokines with a wide variation between individual samples (Fig. S3). The cytokines, CXCL1, CXCL5, and leptin trended towards being higher in the majority of CAFs tested (Fig. 4C), which has also been observed in other studies [43–45]. CXCL1, CXCL5 and leptin resulted in basal cell enrichment in the normal tissue compared to the base with CXCL5 producing the largest change in the normalized JSD score (Figs. 4D-4F and Table S10). However, CXCL1, CXCL5 and leptin did not induce proliferation (Fig. 4G). Therefore, in our studies with tumor tissue the 3D culture was supplemented only with AREG and FGF7.

### TNBC tumor organoids and chemotherapy responses

**Fig. 5:**
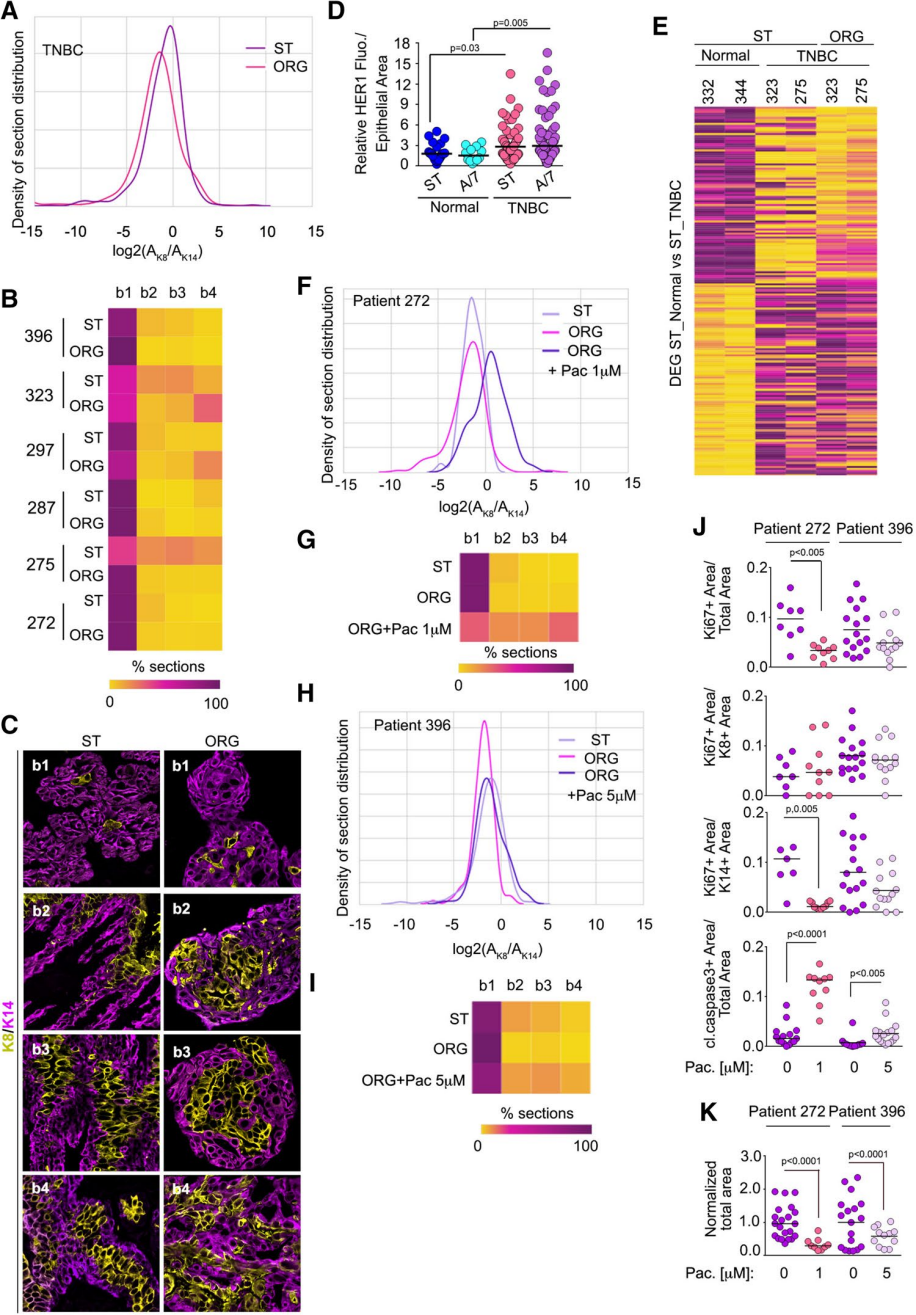
TNBC tissue intra-tumor heterogeneity and drug responses. **A** The probability density distribution for TNBC starting tissue and the resulting organoids. **B** Heat map showing intra- and inter-tumor heterogeneity of all the starting tissue compared to the organoids generated from the tissue. **C** Representative images from ST and resulting organoids. **D** TNBC tumor organoids recapitulate the expression of HER1 observed in the starting tumor tissue. For Normal (N = 24 patients with ≥ 12 organoid sections); for TNBC (N = 6 patients with ≥ 59 organoid sections). **E** Analysis of RNAseq data shows that TNBC tumor tissue and its respective organoids segregate from normal breast tissue. For patients 272 **F**, **G** and 396 **H**, **I** the probability density distribution and heat map analysis of starting tissue and their organoids treated with or without paclitaxel (Pac) is shown. **J** Tumor organoids identify differing intra-tumor proliferation and apoptotic responses to Pac. **K** Inhibition of tumor organoid area in response to PAC as measured by DIC. Area normalized to vehicle control for each patient. See Figure S4 and Tables 3 and 4 and S3

Using the JSD analysis, we next evaluated how effectively the heterogeneity of TNBC tissue could be recapitulated in our in vitro system and whether differences in individual patient response to chemotherapy could be observed. Epithelial fragments were cultured in 3D. Tissues from 6 different patients were obtained; of these one had undergone prior chemotherapy (patient 272), one had been treated with both radiation and chemotherapy (patient 287) and the remaining were therapy naïve. The normalized JSD values obtained from comparing the ST with the organoids ranged from ~ 81 to ~ 33 (Figs. 5A-C and Table 3). The lowest normalized JSD was obtained from patient 275 (Table 3). The reason for the lack of recapitulation of the ST distribution is not obvious as patient 323 had a similar ST distribution to 275 but the organoid culture conditions were able for 323 to capture the phenotypes within the ST as shown by the higher normalized JSD score (Fig. 5B and Table 3). As a further measure of the organoid system to recapitulate the ST, HER1 levels were analyzed and found to be higher in TNBC ST than in normal tissue, consistent with the literature and this difference was maintained in the organoids (Fig. 5D) [46]. Interestingly, addition of EGF instead of AREG would have reduced intra-tumor heterogeneity and generated worse JSD scores (Fig. S2 and Table 2). Additionally, we confirmed that the gene expression differences between TNBC ST and their organoids were similar and differed from normal breast ST (Figs. 5E and S4). Taken together, these data demonstrate that using cellular phenotypes can be used to complement other approaches to assessing whether the organoids recapitulate the ST.

**Table 3:** Individual JSD scores for comparison for starting tissue and organoids generated from corresponding tissue under A7 conditions

	Pat_ID	JSD	JSD_Norm
ER	435	0.33	41.99
ER	461	0.40	28.12
ER	486	0.38	32.14
ER	502	0.33	41.69
ER	549	0.29	49.07
ER	612	0.32	42.41
ER	687	0.16	71.18
TNBC	272	0.11	81.02
TNBC	275	0.37	33.89
TNBC	287	0.13	76.32
TNBC	297	0.22	61.67
TNBC	323	0.23	58.30
TNBC	396	0.15	73.73

A primary goal of recapitulating tumor heterogeneity in vitro is to develop a tractable system for the analysis of drug responses, as tumor recurrence is likely to arise from cells that are unresponsive to the therapy. The JSD method summarizes the effects of proliferation, apoptosis and cell type in a single value that should highlight the development of a treatment -resistant cell population. Therefore, to test the ability of the JSD method to detect resistance, TNBC organoids were treated with paclitaxel, which is frequently used as an initial therapy for patients with TNBC [47]. The dosing and scheduling for paclitaxel was based on the pharmacokinetics observed in patients (Methods). A normalized JSD score of ~ 81 and ~ 74 was obtained with the organoids generated from patients 272 and 396, respectively demonstrating that the culture conditions were able to recapitulate the ST heterogeneity (Table 3). This concordance provides support for the physiological relevance of the drug responses obtained with the organoids. The probability density analysis for patient 272 skews to the right in response to paclitaxel indicating an increase in K8 + cells (Figs. 5F, 5G). The normalized JSD value decreased from ~ 81 with no drug to ~ 20 with a dose of 1 μM paclitaxel (Table 4). To identify a mechanism for changes to the JSD score with paclitaxel, the extent of proliferation and apoptosis was determined. The tumor organoids from patient 272 demonstrated an ~ three-fold decrease in total proliferation, as measured by Ki67, and an ~ eight-fold increase in apoptosis – as measured by cleaved caspase-3 staining (Fig. 5J). Interestingly, the decrease in proliferation was confined to the K14 + population. It was not possible to identify the phenotype of cells that had undergone apoptosis. In agreement with the proliferation and apoptosis data the total organoid area decreased by three-fold (Fig. 5K). Together, these data indicate that the growth of the tumor tissue is sensitive to paclitaxel, which is primarily due to the decreased proliferation of the K14 + population. Importantly, the change in the JSD score readily highlights the development of a therapy-resistant K8 + cell population.

**Table 4:** Individual JSD scores for comparison for TNBC starting tissue and organoids generated from TNBC tissue under varying conditions. See Table S3

Pat_ID	Condition_1	Condition_2	JSD	JSD_Norm
272	A7	PAC 1	0.45	18.87
396	A7	PAC 5	0.22	60.31

In contrast to organoids from patient 272 the probability density for organoids from patient 396 was altered by 5 μM paclitaxel and correspondingly the normalized JSD score decreased by 20% (Figs. 5H, 5I and Table 4). Significant changes in proliferation were not detected but an ~ three-fold increase in apoptosis was observed (Fig. 5J). Consistent with these observations a decrease in organoid size occurred in response to paclitaxel (Fig. 5K). These data show that the growth of the tumor tissues is sensitive to paclitaxel as a result of increased apoptosis. However, the change in the normalized JSD score due to the enrichment in the K8 + population suggests that therapy-resistance may develop.

### Luminal A/B breast cancer organoids and chemotherapy response

**Fig. 6:**
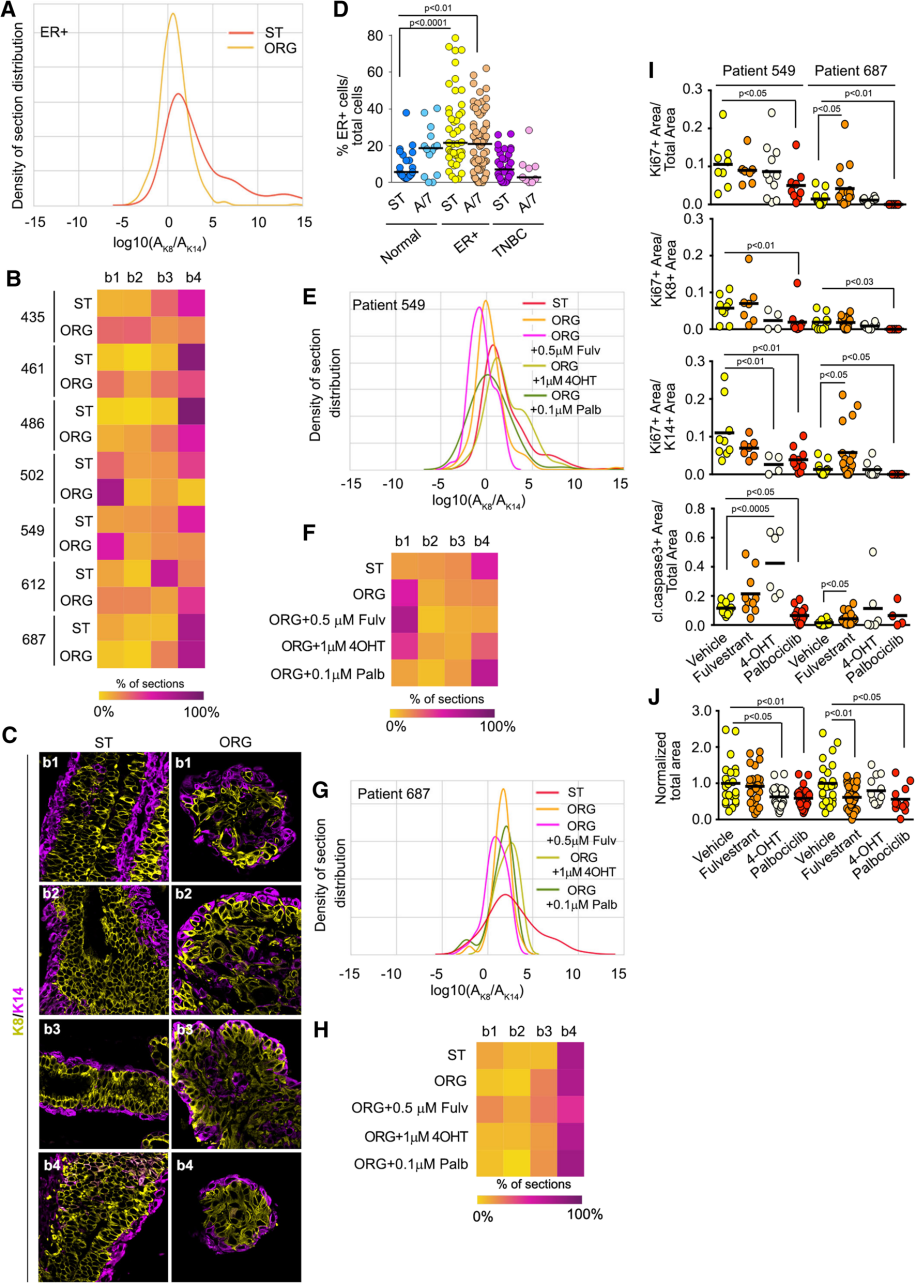
Luminal A/B tissue intra-tumor heterogeneity and drug responses. **A** The probability density distribution for luminal A/B starting tissue and the resulting organoids. **B** Heat map showing intra- and inter-tumor heterogeneity of all the starting tissue compared to the organoids generated from the tissue. **C** Representative images from ST and resulting organoids. **D** Luminal A/B tumor organoids recapitulate the expression of ERα observed in the starting tumor tissue. Data was obtained from ≥ 2 patients and ≥ 9 sections for each analysis. For patients 549 **E**, **F** and 687 **G**, **H** the probability density distribution and heat map analysis of starting tissue and their organoids treated with different chemotherapeutic treatments. **I** Tumor organoids identify differing proliferation and apoptotic responses to chemotherapeutic treatments. **J** Inhibition of tumor organoid area in response to chemotherapeutic treatments as measured by DIC. Area normalized to vehicle control for each patient. See Figures S5 and S6 and Tables 3 and 5 and S3

Tissue from seven different ER + breast cancers were obtained and epithelial fragments from these tumors were cultured in 3D. The normalized JSD values ranged from ~ 28 to ~ 71 (Figs. 6A-C and Table 3). The probability density distributions indicate that we were least successful in generating organoids that are highly enriched for K8 + cells in comparison to the ST. Breast cancers are classified as ER + when > 1% of the cells within the tumor tissue stains for ERα [48]. In analyzing the starting ER + breast tumor tissue in our cohort we found that ~ 20% of the total cells expressed ERα as compared to ~ 5% in normal tissue, which was similar to the distribution obtained in the organoid culture (Fig. 6D). The number of K8 + cells is highly linearly correlated with the number of ERα + cells (Figs. 3B, S5) and therefore, we conclude that organoids with a high percentage of K8 + cells also have a high percentage of ERα + cells. The problem of recapitulating organoids enriched for ERα + cells is consistent with the literature as maintaining ERα expression in culture is known to very difficult [49].

To determine whether we could identify therapy-induced resistance in luminal A/B either anti-estrogens or the CDK4/6 inhibitor were evaluated. In vivo the selective estrogen receptor modulator, tamoxifen, is converted to its active form, 4-hydroxy tamoxifen (4-OHT), and therefore, 4-OHT was used in the in vitro culture. The dosing and scheduling of all drugs was based on the pharmacokinetics observed in patients (Methods).

Organoids were generated from patients 461, 549 and 687. The normalized JSD scores ranged from ~ 28, 49, and 71, respectively (Table 3). Based on limiting amounts of material available from patient 461 only 4-OHT treatments were performed. The heat map shows that 4-OHT resulted in an increase in the K14 + population relative to the ST and the untreated organoid culture, suggesting the potential of developing a treatment-resistant population (Fig. S6 and Tables 3 and 5). The culture conditions were more effective for generating organoids from patient 549 and as a result more extensive analyses could be performed. Palbociclib increased the relative proportion of K8 + cells to more closely resemble the ST (Figs. 6E, 6F and Tables 3 and 5). The increase in the proportion of K8 + cells in the organoids in response to Palbociclib can be explained by the observed decrease in the proliferation of the K14 + population (Fig. 6I). Similar results were observed with 4-OHT with an increase in apoptosis also observed. Thus for patients 461 and 549 the organoid culture conditions promoted the growth of the K14 + population. Interestingly, although 4-OHT and Palbociclib decreased the K14 + population, their effects on the K8 + population were minimal as the proportion of K8 + to K14 + increased with the treatments. These results suggest that the K8 + population in patients 461 and 549 are relatively unaffected by 4-OHT and Palbociclib and may show treatment resistance.

**Table 5:** Individual JSD scores for comparison for ER + starting tissue and organoids generated from ER + tissue under varying conditions. See Tables S2 and S3

Pat_ID	Condition_1	Condition_2	JSD	JSD_Norm
461	A7	4-OHT	0.13	77.60
549	A7	Fulvestrant	0.20	63.22
549	A7	4-OHT	0.12	78.41
549	A7	Palbociclib	0.32	43.59
687	A7	Fulvestrant	0.23	58.17
687	A7	4-OHT	0.06	88.65
687	A7	Palbociclib	0.11	80.28

In contrast to patients 461 and 549 the culture conditions for patient 687 were able to recapitulate the ST to a much greater degree as shown by the normalized JSD score (Figs. 6G, 6H and Table 3). These results are surprising given that 687 is also enriched in K8 + cells as are 461 and 549. These results demonstrate the difficulties in recapitulating the ST as individual patient tumor tissues, even if they are the same tumor type, respond differently to the same culture conditions. For organoids generated from 687 the selective estrogen degrader, Fulvestrant, resulted in a shift towards the left indicating an increase in the K14 + population with the JSD score decreasing from ~ 71 to ~ 58 (Table 5). Fulvestrant increased apoptosis by ~ three-fold but unexpectedly increased proliferation in the K14 + population by ~ five-fold (Fig. 6I). This change in the normalized JSD score provides a readout for the development of a K14 + resistant population, which may be important for therapy response despite overall reduced growth of the tumor. No effect on proliferation, apoptosis or organoid size was observed with 4-OHT. Palbociclib reduced both K8 + and K14 + cell proliferation and consistent with these observations organoid size was decreased (Figs. 6I and 6J) [50]. Importantly, Palbociclib treatment for this patient tissue did not substantially change the distribution or normalized JSD score, and therefore, might be an effective therapy for this patient. Taken together, these results demonstrate that the analysis of K8 and K14 distribution appears sufficient to identify differences between chemotherapeutic responses in individual patients and thus, may provide a basis for use in personalized medicine.

## Discussion

Breast cancer organoid models have been proposed for use in personalized medicine and in the identification of novel therapeutic targets. A significant challenge for this technology is the recapitulation of the starting tissue and its intrinsic heterogeneity into the organoid model in order to increase the likelihood of obtaining physiologically relevant information. This intrinsic heterogeneity occurs through cell-autonomous and non-cell autonomous mechanisms, which are important to capture as it contributes to therapeutic response [51–54]. To address the need for an easily managed method for evaluating whether organoids recapitulate the tumor in situ we describe an approach that generates a quantitative readout. This method is based on imaging data that provides an objective analysis of the similarity between organoids and the source tissue, and between different culture conditions. This approach can also facilitate the analysis of modifications to the culture conditions, such as the addition of immune cells or adipocytes or drug treatments. The method complements omics approaches by validating that the culture conditions are permissive for the activation of the relevant signal transduction pathways, which impact cellular phenotype.

In our example, distribution curves based on the frequency of K8 and K14 were generated from ≥ 23 organoid and starting tissue sections for each sample to obtain an 0.85 confidence level that the heterogeneity within the tissue had been captured. The similarity of these distributions was evaluated using the JSD method, which provided a quantitative value. For ease of use, the method described uses only two standard breast biomarkers, K8 and K14. However, markers other than K8 and K14 could be used and the choice would depend on the study focus. Markers in addition to K8 and K14 could be included although the complexity of the analysis will increase. For example, comparing RNA-seq data of starting tissue with organoids required an additional metric beyond the JSD analysis [55] as the JSD approach could only satisfy the distribution evaluation criteria and not expression level differences. These additional complications do not occur in our data using only the distribution of two markers.

The JSD method summarizes the effects of proliferation, apoptosis and differentiation in a single value providing an evaluation of organoid fidelity and therapy-resistant cell populations. For example, in ER + breast cancer organoids generated from patient 687, the decrease in the normalized JSD score by fulvestrant indicates that the tissue heterogeneity is being altered. Analysis of the bin distribution profile that is used to generate the normalized JSD score shows enrichment of a K14 + population, which could be a possible source of resistance. In contrast Palbociclib does not change the normalized JSD score, and the lack of a resistant population suggests that Palbociclib would be an effective treatment for this patient as it also reduced tumor growth. The JSD method also provides a simple readout to identify culture conditions that influence tissue development as seen in particular for CXCL5. Addition of CXCL5 increased the proportion of K14 + cells in normal tissue and K14 + cells have been implicated in metastatic progression [56].

Analysis of cytokines and growth factors secreted by normal and CAFs demonstrated the variability inherent between patients although HER1 and FGFR ligands were detected in all samples. Previously, HER1 and FGFR ligands were necessary for the in vitro development of normal breast [13], and in this study were sufficient to recapitulate the majority of TNBCs in 3D culture with high fidelity. However, the HER1 and FGFR cocktail was only partially successful in recapitulating breast cancer tissue highly enriched for ERα cells. In part this problem may be due to the Matrigel, the matrix used in this study, which is considered a soft material. Matrix stiffness has been found to be important in maintaining ERα in breast cancer cells but further research is needed [49]. The ability to propagate the inherent heterogeneity with HER1 and FGFR for both TNBC and ER + breast cancer suggests that there are intrinsic differences in how individual cells within these tumors respond to the signaling pathways. Our observations may partially explain the lack of success of HER1 and FGFR inhibitors in breast cancer, as those cells that are less dependent on HER1 or FGFR may generate resistance [57, 58].

Evaluating the similarity between complex systems is extremely challenging and we have demonstrated the utility of the JSD approach to provide a quantitative measure that is particularly useful for personalized medicine. Standard statistical approaches are not applicable in comparing organoids to the ST as the sample size is one. The importance of analyzing each patient tissue separately was most effectively demonstrated when evaluating the ER + breast cancer patient samples 461, 549 and 687. In the same culture media the K14 + population expanded relative to the K8 + population in organoids generated from patients 461 and 549; however, these results were not observed with organoids generated from patient 687. This difference is most likely due to genomic alterations that result in the activation of signaling pathways that generate divergent responses in the patient’s tissue. These results also highlight the importance of a personalized medicine approach in identifying the best treatment option for the patient.

A major issue in translating organoid-based data into the clinic is the absence of a threshold response [59]. The analysis based on the JSD approach could provide such a quantitative readout to aid in identifying the best treatment options for a particular patient. For this approach to be successful it would be necessary to validate that the JSD approach can be used as a predictor of patient outcome by comparing the results obtained from treated organoids to the patient’s response. The data acquisition and analysis can readily be automated making the JSD approach suitable for translation.

## Methods

### Organoid and fibroblast isolation

Human breast tissue from reduction mammoplasty or breast cancer surgery was collected as waste tissue with institutional review board approval. A list of age, race, and diagnosis for each patient used in this study is provided (Table S1). Organoids were prepared as previously described [13]. Briefly, tissue was minced and digested in Collagenase A medium (DMEM/F12 (Thermo Fisher Scientific), 1 mg/mL Collagenase A (Roche Diagnostics), 1 μg/mL insulin (Sigma-Aldrich), 600 U/μL Nystatin (Sigma-Aldrich), 100 U/mL penicillin–100 μg/mL streptomycin (Thermo Fisher Scientific)) for 18-21 h in a 37 °C 5% CO_2_ incubator. Digested material was pelleted at 180 g for 5 min and the supernatant collected for fibroblast isolation. The remaining pellet was resuspended in DMEM/F12 with DNAse I (1000 U/ml) (Sigma-Aldrich) for 3–5 min in a 37°C 5% CO_2_ incubator. Fetal bovine serum (FBS) (0.5 mL) was added, and the digested tissue was pelleted at 180 g for 10 min. The pellet was resuspended in 9 ml of DMEM/F12 and centrifuged at 350 g for 15 s. This wash was repeated 4–6 times. The pellet was resuspended in 1 ml of base medium (DMEM/F12, 1 μg/mL hydrocortisone (Sigma-Aldrich), 10 μg/mL insulin-5.5 μg/mL transferrin–6.7 ng/mL selenium-2 μg/mL ethanolamine (Thermo Fisher Scientific), 2.5 μg/mL Amphotericin B (Sigma-Aldrich), 50 μg/mL gentamicin (Thermo Fisher Scientific), 100 U/mL penicillin-100 μg/mL streptomycin). A volume of 60 μl of a 60% Matrigel in base media was added into the wells of an 8-well LabTek plate and solidified for 15 min in a 37°C 5% CO_2_ incubator. Organoids were counted and resuspended in a 50% Matrigel in base media. A volume of 40 μl of Matrigel/organoid solution containing 30–40 medium sized organoids was plated onto the solidified Matrigel layer and allowed to solidify for 15 min at 37°C.

### Organoid culture and drug treatment

Drug treatments starting at day 0 were treated with 4-hydroxy tamoxifen (Sigma-Aldrich), fulvestrant (Santa Cruz Biotechnology Inc.), palbocicilib (Selleckchem), or paclitaxel (R&D systems). For all conditions, the medium was replaced every 24–48 h and drugs were added with fresh medium every 24–48 h as appropriate. Paclitaxel has a half-life of 27 h (https://reference.medscape.com/drug/taxol-paclitaxel-342187#10); the active metabolites of tamoxifen have half-lives of ~ 17 h [60]; fulvestrant has a half-life of 40 d (https://reference.medscape.com/drug/faslodex-fulvestrant-342224#10); and palbociclib has a half-life of ~ 29 h (https://reference.medscape.com/drug/ibrance-palbociclib-999995#10). The concentrations of drugs chosen were based on the maximum serum level in patients. The organoids were cultured for 7–10 days.

### Immunostaining

Organoids were fixed and immunostained as previously described [13]. Detailed methods for immunostaining, imaging, and analysis are provided in the supplementary experimental procedures.

### Data processing

Organoids on average have smaller area than ST. Therefore, to correct for potential artifacts in K8/K14 ratio, we subdivided the images obtained from ST into smaller tiles to match an average organoid size. K8/K14 quantitation was carried out on the resulting tiles if the total tissue area per image was greater than 5% of image area. K8 and K14 area were measured and log2 of K8/K4 ratio calculated. Median and quartile values of log2(K8/K14) from ST of normal breast tissue were determined and set as bin boundaries: -inf:Q25, Q25-median, median-Q75, Q75-inf. Subsequently all images were classified in these bins, and bin distribution was calculated per condition (N of sections in the bin_(1–4)_/Total N of sections per condition) to generate distribution table. These distributions were then used to calculate JSD values for comparison of any two given conditions.

Quantitation and data analysis was carried out in Python3.8.10 using the following packages: Pillow (image tilling and RGB quantitation); Pandas, NumPy, Matplotlib and SciPy (data management, data manipulation, and statistical analysis); Seaborn (data visualization).

### Conditioned media

Fibroblasts obtained during the isolation of epithelial clusters were plated in base medium with 10% FBS. After two passages, the fibroblasts were washed extensively and cultured in base medium without serum. Conditioned medium was collected after 48 h. For conditioned medium from organoids, epithelial clusters were cultured in base media and medium was collected every 48 h for 6 days. The conditioned medium from fibroblasts and organoids was analyzed using Human Cytokine Antibody Array C5 (RayBiotech, Inc.). Multianalyte profiling of fibroblast-conditioned media was performed by the Vanderbilt Hormone Assay and Analytical Services Core using the Luminex-100 system.

### RNA-sequencing

RNA samples were prepared using the TruSeq mRNA library method (poly-A selected). Sequencing was done using the Illumina HiSeq 3000 at 2X75 paired-end reads by Vanderbilt Technologies for Advanced Genomics with a mean of 30e6 reads per library. TopHat (v2.0.9) spliced aligner software was used to align reads to hg19, using refseq transcripts as a guide [61]. Transcripts were assembled and quantified using refseq transcripts as a guide with cufflinks, and normalized FPKMs generated using cuffnorm [62]. Normalized FPKM expression levels were analyzed in R/Bioconductor. Principle Component Analysis was performed using pcaMethods [63].

### Statistical analysis

Statistical analyses were performed using GraphPad Prism 6. Statistical significance was determined using the Mann–Whitney test (two-sided) and all p-values < 0.05 are reported.

## Supplementary Information

Below is the link to the electronic supplementary material.

Supplementary file1 (PDF 9175 KB)

## Data Availability

All data generated or analyzed are available upon request.
