# Supplementary material for: Identifying the effectiveness of 3D culture systems to recapitulate breast tumor tissue in situ
Source: Cell Oncol (Dordr). 2023 Sep 30;47(2):481–96. doi: 10.1007/s13402-023-00877-8 (PMC11090829; doi:10.1007/s13402-023-00877-8)
Supplement: Supplementary file 1 — Supplementary file1 (PDF 9175 KB) [file 13402_2023_877_MOESM1_ESM.pdf]

Table S1. Patient Information. See Figure 1

Patient ID	Age	Race	Diagnosis	# of sections analysed
494	64	Caucasian	Normal	100
506	62	Caucasian	Normal	162
520	35	Caucasian	Normal	51
552	42	Caucasian	Normal	286
581	43	African-American	Normal	290
586	32	African-American	Normal	128
677	40	Caucasian	Normal	128
272	47	African-American	TNBC	88
275	28	Caucasian	TNBC	222
287	56	African-American	TNBC	66
297	56	African-American	TNBC	55
323	43	Caucasian	TNBC	24
396	40	African-American	TNBC	26
435	58	Caucasian	ER+	330
461	50	Caucasian	ER+	87
486	30	Caucasian	ER+	41
502	62	Caucasian	ER+	50
549	54	Caucasian	ER+	23
612	68	Caucasian	ER+	36
628	44	Caucasian	ER+	26
658	49	Caucasian	ER+	41
674	40	Caucasian	ER+	49
679	52	Caucasian	ER+	23
687	45	Caucasian	ER+	125
707	52	African-American	ER+	29
711	35	Caucasian	ER+	46
**Median age:**	**47**		**SUM**	**2532**

Table S2. Bin distribution of starting tissue sections. See Figure 1 and Table 1

Patient ID	Condition	# of Sec. In Bin 1	# of Sec. In Bin 2	# of Sec. In Bin 3	# of Sec. In Bin 4	Sum
272	ST	92	7	1	0	100
275	ST	66	31	36	29	162
287	ST	48	0	0	3	51
297	ST	247	21	9	9	286
323	ST	152	52	55	31	290
396	ST	109	10	7	2	128
435	ST	12	13	38	65	128
461	ST	4	0	7	77	88
486	ST	4	1	11	206	222
494	ST	14	26	16	10	66
502	ST	16	8	9	22	55
506	ST	18	3	2	1	24
520	ST	8	7	6	5	26
549	ST	50	55	63	162	330
552	ST	6	15	26	40	87
581	ST	14	13	7	7	41
586	ST	17	16	17	0	50
612	ST	3	1	14	5	23
628	ST	0	1	2	33	36
656	ST	5	2	13	6	26
674	ST	9	6	5	21	41
677	ST	9	6	11	23	49
679	ST	0	1	5	17	23
687	ST	16	9	9	91	125
707	ST	8	3	6	12	29
711	ST	0	1	7	38	46
**Normal**		**86**	**86**	**85**	**86**	**343**
**ER+**		**127**	**101**	**189**	**755**	**1172**
**TNBC**		**714**	**121**	**108**	**74**	**1017**
					**SUM**	**2532**

Table S3. Bin distribution of organoid sections. See Figures 4, 5, 6 and Table 3

Patient ID	Condition	# of Sec. In Bin 1	# of Sec. In Bin 2	# of Sec. In Bin 3	# of Sec. In Bin 4	Sum
272	A7	174	6	2	4	186
275	A7	204	10	4	4	222
287	A7	115	4	0	2	121
297	A7	67	4	10	15	96
323	A7	97	13	19	51	180
396	A7	121	1	2	0	124
435	A7	18	23	13	8	62
461	A7	28	11	28	29	96
486	A7	14	24	31	53	122
494	A7	28	11	21	23	83
502	A7	46	3	10	2	61
520	A7	96	36	37	9	178
549	A7	84	21	33	25	163
552	A7	69	33	35	13	150
581	A7	12	2	3	0	17
586	A7	17	7	4	14	42
612	A7	4	2	3	5	14
687	A7	3	2	12	23	40
494	Base	4	3	8	17	32
502	Base	8	1	5	5	19
520	Base	14	14	7	0	35
552	Base	18	20	14	5	57
581	Base	13	9	4	2	28
586	Base	9	1	9	11	30
494	E7	7	7	10	12	36
581	E7	23	1	2	0	26
586	E7	24	0	0	1	25
581	CXCL5	7	5	3	1	16
586	CXCL5	7	0	1	0	8
494	CXCL1	7	9	6	7	29
520	CXCL1	6	8	8	3	25
552	CXCL1	7	11	11	7	36
581	CXCL1	3	0	7	2	12
502	R-s	6	2	4	1	13
520	R-s	13	0	0	0	13
552	R-s	36	7	4	2	49
549	Fulvestrant	41	1	9	4	55
687	Fulvestrant	10	8	12	15	45
581	Leptin	5	3	5	0	13
586	Leptin	5	1	2	1	9
549	4-OHT	28	10	8	17	63
461	4-OHT	30	6	17	13	66
687	4-OHT	1	1	3	8	13
272	PAC 1	66	16	21	12	115

396	PAC 1	166	1	0	0	167
396	PAC 5	124	12	12	8	156
549	Palbociclib	15	7	29	44	95
687	Palbociclib	1	1	3	12	17

Table S4. Individual JSD scores for comparisons between starting tissues of each ER+ patient (ST_ER_Pat1) and each TNBC patient (ST_TNBC_Pat2).

ST_ER_Pat1	ST_TNBC_Pat2	JSD	JSD_Norm
435	272	0.65	-16.56
435	275	0.31	44.06
435	287	0.65	-15.46
435	297	0.59	-5.95
435	323	0.41	27.24
435	396	0.60	-6.70
461	272	0.76	-36.41
461	275	0.53	5.70
461	287	0.70	-25.51
461	297	0.71	-27.59
461	323	0.61	-9.54
461	396	0.72	-28.96
486	272	0.78	-39.79
486	275	0.58	-3.44
486	287	0.73	-30.88
486	297	0.74	-31.80
486	323	0.66	-17.51
486	396	0.75	-34.64
502	272	0.49	13.04
502	275	0.12	78.69
502	287	0.49	12.24
502	297	0.42	25.18
502	323	0.22	59.94
502	396	0.42	24.36
549	272	0.60	-7.49
549	275	0.26	53.54
549	287	0.60	-7.47
549	297	0.54	3.50
549	323	0.35	36.73
549	396	0.55	1.80
612	272	0.65	-15.25
612	275	0.34	39.11
612	287	0.66	-17.96
612	297	0.59	-5.85
612	323	0.40	29.38
612	396	0.58	-3.65
628	272	0.80	-42.19
628	275	0.57	-1.23
628	287	0.76	-36.42
628	297	0.75	-33.80
628	323	0.65	-15.28
628	396	0.76	-36.37

658	272	0.58	-2.75
658	275	0.26	52.96
658	287	0.62	-11.35
658	297	0.52	6.69
658	323	0.29	48.22
658	396	0.51	8.39
674	272	0.56	0.67
674	275	0.19	66.00
674	287	0.58	-2.89
674	297	0.49	11.95
674	323	0.27	51.11
674	396	0.50	10.40
679	272	0.80	-42.21
679	275	0.52	6.89
679	287	0.77	-37.12
679	297	0.75	-33.08
679	323	0.61	-8.30
679	396	0.75	-33.95
687	272	0.65	-16.31
687	275	0.37	33.36
687	287	0.63	-12.35
687	297	0.60	-6.25
687	323	0.46	17.38
687	396	0.61	-8.80
707	272	0.54	3.76
707	275	0.20	64.63
707	287	0.53	5.41
707	297	0.47	15.37
707	323	0.30	46.30
707	396	0.48	14.22
711	272	0.80	-43.62
711	275	0.54	3.26
711	287	0.77	-36.92
711	297	0.75	-34.68
711	323	0.63	-11.91
711	396	0.76	-35.53
	**Average:**	**0.56**	**-0.13**

Table S5. Individual JSD scores for comparisons between each starting normal breast tissue samples (ST_N_Pat1) and (ST_N_Pat2).

ST_N_Pat1	ST_N_Pat2	JSD	JSD_Norm
494	506	0.39	29.63
494	515	0.59	-5.27
494	520	0.16	71.20
494	552	0.30	47.27
494	581	0.20	64.28
494	586	0.18	67.82
494	669	0.37	33.30
494	677	0.30	45.72
506	515	0.75	-33.15
506	520	0.34	39.27
506	552	0.57	-1.09
506	581	0.26	54.54
506	586	0.27	51.38
506	669	0.54	3.10
506	677	0.50	10.07
520	552	0.26	52.87
520	581	0.10	82.48
520	586	0.24	57.36
520	669	0.28	49.92
520	677	0.22	61.12
552	581	0.35	36.79
552	586	0.43	23.39
552	669	0.16	72.22
552	677	0.14	75.85
581	586	0.21	62.20
581	669	0.35	36.99
581	677	0.30	45.88
586	669	0.47	15.41
586	677	0.41	27.34
	**Average:**	**0.33**	**40.62**

Table S6. Individual JSD scores for comparisons between each starting ER+ breast cancer tissue samples (ST_ER_Pat1) and (ST_ER_Pat2).

ST_ER_Pat1	ST_ER_Pat2	JSD	JSD_Norm
435	461	0.30	45.96
435	486	0.37	33.93
435	502	0.20	63.51
435	549	0.09	83.35
435	612	0.28	50.81
435	628	0.34	38.92
435	656	0.31	44.72
435	674	0.17	69.14
435	679	0.26	54.52
435	687	0.18	68.36
435	707	0.15	74.18
435	711	0.28	49.56
461	486	0.15	72.59
461	502	0.43	23.06
461	549	0.33	40.99
461	612	0.51	8.45
461	628	0.20	64.25
461	656	0.58	-2.69
461	674	0.43	23.33
461	679	0.20	64.08
461	687	0.22	60.73
461	707	0.36	36.35
461	711	0.17	69.40
486	502	0.49	12.15
486	549	0.38	32.25
486	612	0.60	-6.43
486	628	0.12	79.01
486	656	0.64	-14.48
486	674	0.48	14.88
486	679	0.24	57.93
486	687	0.24	56.92
486	707	0.42	25.14
486	711	0.21	62.30
502	549	0.16	71.43
502	612	0.29	47.92
502	628	0.48	14.43
502	656	0.26	53.42
502	674	0.13	77.00
502	679	0.42	24.62
502	687	0.28	50.46
502	707	0.08	85.08
502	711	0.44	20.97

549	612	0.32	42.18
549	628	0.36	36.45
549	656	0.31	43.90
549	674	0.11	79.99
549	679	0.31	45.11
549	687	0.15	72.56
549	707	0.10	81.63
549	711	0.33	40.65
612	549	0.32	42.18
612	628	0.57	-1.48
612	656	0.17	69.91
612	674	0.30	46.56
612	679	0.45	20.40
612	687	0.44	22.05
612	707	0.31	45.03
612	711	0.47	15.66
628	656	0.61	-8.61
628	674	0.45	19.81
628	679	0.17	69.60
628	687	0.24	57.37
628	707	0.41	26.93
628	711	0.16	71.28
658	376	0.64	-14.18
658	674	0.24	57.50
658	679	0.51	8.31
658	687	0.45	19.15
658	707	0.31	45.24
658	711	0.54	2.90
674	679	0.40	29.14
674	687	0.26	54.02
674	707	0.14	75.32
674	711	0.43	24.19
679	687	0.25	55.69
679	707	0.36	36.62
679	711	0.05	91.45
687	707	0.20	64.03
687	711	0.26	53.81
707	711	0.37	33.32
	**Average:**	**0.32**	**43.48**

Table S7. Individual JSD scores for comparisons between starting tissues of each individual ER+ patient (ST_ER_Pat1) and each normal breast tissue samples (ST_N_Pat2).

ST_ER_Pat1	ST_N_Pat2	JSD	JSD_Norm
435	494	0.33	40.89
435	506	0.54	3.19
435	520	0.30	46.88
435	552	0.08	86.08
435	581	0.34	38.55
435	586	0.49	11.92
435	677	0.10	81.43
461	494	0.57	-2.27
461	506	0.68	-21.76
461	520	0.53	4.55
461	552	0.36	36.27
461	581	0.56	0.23
461	586	0.72	-28.95
461	677	0.34	39.75
486	494	0.60	-7.45
486	506	0.71	-27.46
486	520	0.57	-1.59
486	552	0.38	31.45
486	581	0.59	-5.35
486	586	0.75	-34.81
486	677	0.38	32.85
502	494	0.26	54.25
502	506	0.38	32.40
502	520	0.18	68.52
502	552	0.22	60.71
502	581	0.20	63.82
502	586	0.41	25.91
502	677	0.10	81.90
549	494	0.27	51.22
549	506	0.48	14.06
549	520	0.23	58.39
549	552	0.12	78.68
549	581	0.27	52.42
549	586	0.46	17.91
549	677	0.06	89.74
612	494	0.36	35.61
612	506	0.53	6.09
612	520	0.33	41.12
612	552	0.27	51.16
612	581	0.39	30.82
612	586	0.42	24.51
612	677	0.29	48.81

628	494	0.59	-5.83
628	506	0.72	-29.23
628	520	0.57	-1.11
628	552	0.37	33.92
628	581	0.59	-4.84
628	586	0.75	-34.09
628	677	0.38	32.36
658	494	0.29	47.76
658	506	0.46	18.18
658	520	0.25	55.81
658	552	0.24	57.56
658	581	0.31	45.21
658	586	0.37	33.64
658	677	0.22	59.97
674	494	0.30	46.07
674	506	0.44	20.85
674	520	0.24	56.54
674	552	0.20	63.77
674	581	0.26	52.98
674	586	0.48	14.86
674	677	0.10	82.64
679	494	0.51	8.87
679	506	0.69	-23.07
679	520	0.49	12.82
679	552	0.25	54.71
679	581	0.52	6.50
679	586	0.66	-18.55
679	677	0.30	46.74
687	494	0.44	21.50
687	506	0.56	0.39
687	520	0.39	29.79
687	552	0.26	53.58
687	581	0.41	26.37
687	586	0.61	-8.17
687	677	0.20	64.11
707	494	0.28	49.58
707	506	0.40	28.78
707	520	0.20	63.94
707	552	0.21	63.22
707	581	0.24	57.30
707	586	0.43	23.71
707	677	0.08	86.10
711	494	0.55	0.98
711	506	0.71	-26.49
711	520	0.53	5.66

711	552	0.31	44.83
711	581	0.56	0.21
711	586	0.71	-26.30
711	677	0.33	40.42
	**Average:**	**0.40**	**29.09**

Table S8. Individual JSD scores for comparisons between starting tissues of each individual TNBC sample (ST_TNBC_Pat) and (ST_TNBC_Pat2).

ST_TNBC_Pat1	ST_TNBC_Pat2	JSD	JSD_Norm
272	275	0.40	28.56
272	287	0.22	60.78
272	297	0.11	81.31
272	323	0.34	40.21
272	396	0.11	80.64
275	287	0.43	23.35
275	297	0.33	41.39
275	323	0.11	80.66
275	396	0.33	40.49
287	297	0.20	63.90
287	323	0.40	28.69
287	396	0.23	59.23
297	287	0.20	63.90
297	323	0.27	52.53
297	396	0.07	87.71
323	396	0.27	52.37
	**Average:**	**0.25**	**55.36**

Table S9. Individual JSD scores for comparisons between starting tissues of each individual TNBC patient (ST_TNBC_Pat1) and each ER+ tissue samples (ST_ER+_Pat2).

ST_TNBC_Pat1	ST_N_Pat2	JSD	JSD_Norm
272	494	0.55	2.07
272	506	0.20	64.65
272	520	0.49	12.48
272	552	0.69	-22.83
272	581	0.46	17.33
272	586	0.46	17.91
272	677	0.60	-7.23
275	494	0.19	66.89
275	506	0.26	53.23
275	520	0.08	85.14
275	552	0.32	43.08
275	581	0.11	81.22
275	586	0.28	50.12
275	677	0.24	56.59
287	494	0.60	-6.47
287	506	0.28	50.06
287	520	0.53	6.21
287	552	0.69	-23.12
287	581	0.51	9.58
287	586	0.57	-1.41
287	677	0.59	-5.63
297	494	0.48	13.47
297	506	0.11	80.40
297	520	0.42	25.48
297	552	0.62	-10.96
297	581	0.39	30.07
297	586	0.43	22.79
297	677	0.53	5.59
323	494	0.24	56.87
323	506	0.17	68.77
323	520	0.16	71.29
323	552	0.41	27.63
323	581	0.15	72.66
323	586	0.26	53.50
323	677	0.33	41.48
396	494	0.47	15.42
396	506	0.09	83.23
396	520	0.41	27.01
396	552	0.62	-10.32
396	581	0.39	31.23
396	586	0.40	28.81
396	677	0.53	5.59

	**Average:**	**0.39**	**30.71**

Table S10 Normalized JSD scores for comparison between normal starting tissue and organoids generated from normal tissue under varying conditions. Condition_1: starting tissue; Condition_2: organoids culture condition.

Condition_1	Condition_2	JSD	JSD_Norm
ST	A7	0.11	80.36
ST	CXCL5	0.24	57.14
ST	CXCL1	0.15	73.21
ST	Leptin	0.15	73.21

Supplementary Figure Legends

Supplementary Figure Legends

Figure S1 Representative sections stained with hematoxylin and eosin stain of patient samples used in this study. Human breast tissue from reduction mammoplasty or breast cancer surgery was collected as waste tissue with institutional review board approval. See Figure 1

Figure S2 The probability density distribution for TNBC starting tissue and the resulting organoids cultured under various conditions. Base media was supplemented with AREG and FGF-7 (A/7), EGF and FGF-7 (E/7). See Figures 2, 4 and 5

Figure S3 Analysis of supernatants obtained from normal, or cancer associated fibroblasts (CAFs). The circles indicate analysis from normal fibroblasts and the squares from CAFs. The analysis for each factor analyzed are presented side by side. The readout for each factor was normalized to positive and negative controls. Individual points were obtained from analysis of fibroblasts obtained from different patients. See Figure 4

Figure S4 Cumulative Z-scores calculated based on normalized counts across genes differentially expressed between TNBC ST and Normal ST. See Figure 5

Figure S5 A positive linear correlation was observed for the number of K8+ versus ERα+ cells in ER+ breast cancer organoids. Correlation r values for each patient are calculated. See Figure 6

Figure S6 (A) Probability density distribution and (B) heat map analysis of Patient 461 tumor organoid responses to drug treatment. See Figure 6.

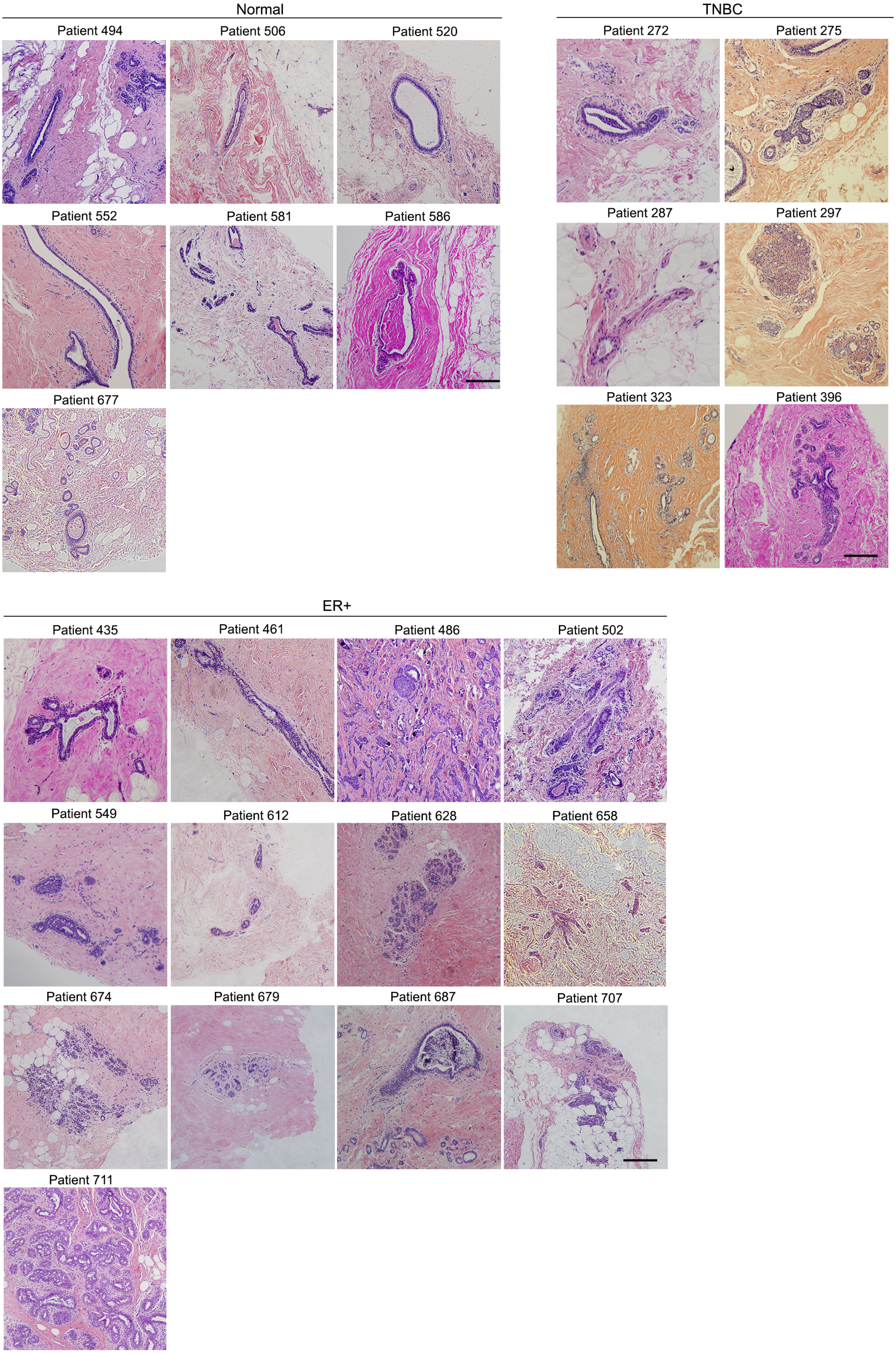
Normal
Patient 494
Patient 506
Patient 520
Patient 552
Patient 581
Patient 586
Patient 677
TNBC
Patient 272
Patient 275
Patient 287
Patient 297
Patient 323
Patient 396
ER+
Patient 435
Patient 461
Patient 486
Patient 502
Patient 549
Patient 612
Patient 628
Patient 658
Patient 674
Patient 679
Patient 687
Patient 707
Patient 711

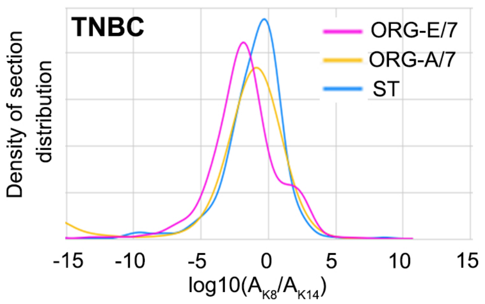
TNBC
ORG-E/7
ORG-A/7
ST
Density of section distribution
-15
-10
-5
0
5
10
15
log10(A_K8/A_K14)

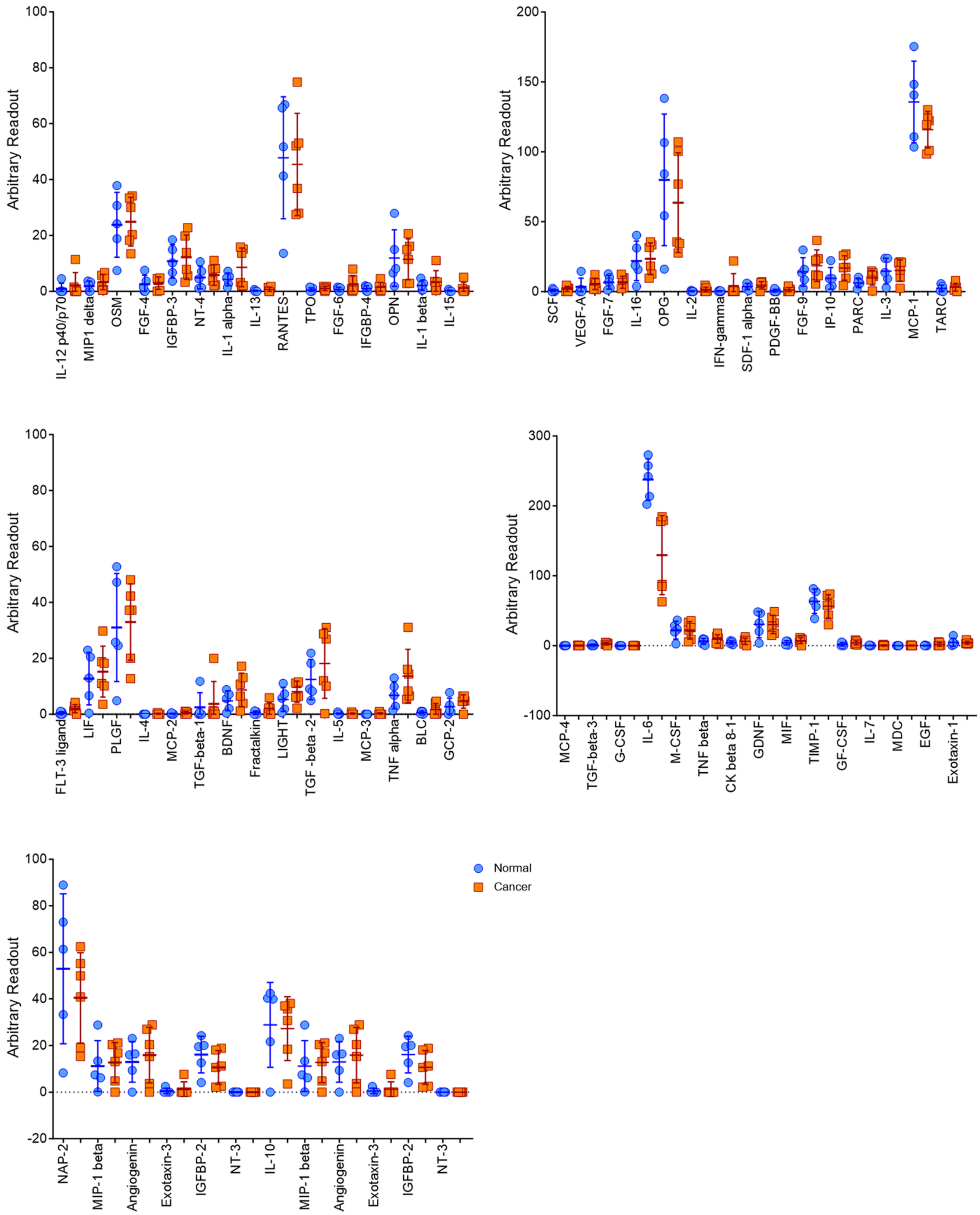

Arbitrary Readout
IL-12 p40/p70
MIP1 delta
OSM
FGF-4
IGFBP-3
NT-4
IL-1 alpha
IL-13
RANTES
TPO
FGF-6
IFGBP-4
OPN
IL-1 beta
IL-15
SCF
VEGF-A
FGF-7
IL-16
OPG
IL-2
IFN-gamma
SDF-1 alpha
PDGF-BB
FGF-9
IP-10
PARC
IL-3
MCP-1
TARC
FLT-3 ligand
LIF
PLGF
IL-4
MCP-2
TGF-beta-1
BDNF
Fractalkin
LIGHT
TGF -beta -2
IL-5
MCP-3
TNF alpha
BLC
GCP-2
MCP-4
TGF-beta-3
G-CSF
IL-6
M-CSF
TNF beta
CK beta 8-1
GDNF
MIF
TIMP-1
GF-CSF
IL-7
MDC
EGF
Exotaxin-1
NAP-2
MIP-1 beta
Angiogenin
Exotaxin-3
IGFBP-2
NT-3
IL-10
Normal
Cancer

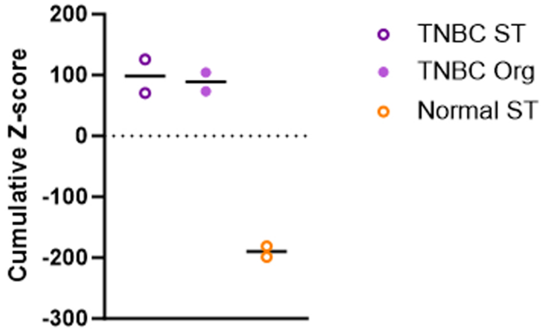
200
100
0
-100
-200
-300
Cumulative Z-score
TNBC ST
TNBC Org
Normal ST

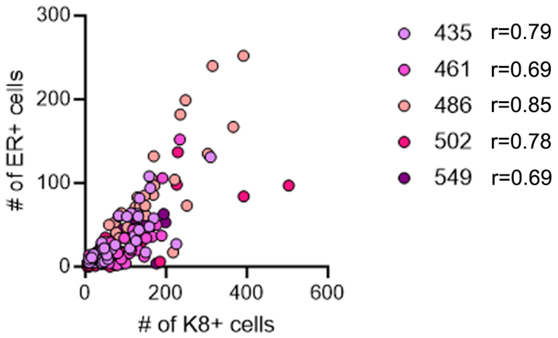
300
200
100
0
# of ER+ cells
0
200
400
600
# of K8+ cells
435 r=0.79
461 r=0.69
486 r=0.85
502 r=0.78
549 r=0.69

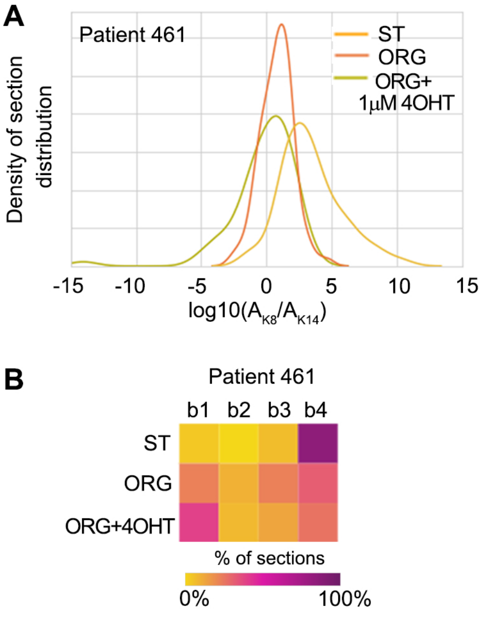
A
Patient 461
ST
ORG
ORG+
1μM 4OHT
Density of section distribution
-15
-10
-5
0
5
10
15
log10(AK8/AK14)
B
Patient 461
b1
b2
b3
b4
ST
ORG
ORG+4OHT
% of sections
0%
100%
